# Burden of neurologic diseases in BRICS countries (1990 to 2021): an analysis of 2021 Global Burden of Disease Study

**DOI:** 10.3389/fneur.2024.1500551

**Published:** 2024-12-05

**Authors:** Shubham Chauhan, Shilpa Gaidhane, G. Padma Priya, Pawan Sharma, Mahakshit Bhat, Shilpa Sharma, M. Ravi Kumar, Aashna Sinha, Quazi Syed Zahiruddin, Navneet Dev, Ganesh Bushi, Diptismita Jena, Muhammed Shabil, Sanjit Sah, Rukshar Syed, Kamal Kundra, Alisha Dash, Shailesh Kumar Samal

**Affiliations:** ^1^Saveetha Medical College and Hospital, Saveetha Institute of Medical and Technical Sciences, Saveetha University, Chennai, India; ^2^One Health Centre (COHERD), Jawaharlal Nehru Medical College, Datta Meghe Institute of Higher Education, Wardha, India; ^3^Department of Chemistry and Biochemistry, School of Sciences, JAIN (Deemed to be University), Bangalore, Karnataka, India; ^4^Department of Sciences, Vivekananda Global University, Jaipur, Rajasthan, India; ^5^Department of Medicine, National Institute of Medical Sciences, NIMS University Rajasthan, Jaipur, India; ^6^Chandigarh Pharmacy College, Chandigarh Group of Colleges-Jhanjeri, Mohali, Punjab, India; ^7^Department of Chemistry, Raghu Engineering College, Visakhapatnam, Andhra Pradesh, India; ^8^Uttaranchal Institute of Pharmaceutical Sciences, Division of Research and Innovation, Uttaranchal University, Dehradun, India; ^9^South Asia Infant Feeding Research Network (SAIFRN), Division of Evidence Synthesis, Global Consortium of Public Health and Research, Datta Meghe Institute of Higher Education, Wardha, India; ^10^Department of Dermatology, Graphic Era Deemed to be University, Dehradun, India; ^11^School of Pharmaceutical Sciences, Lovely Professional University, Phagwara, India; ^12^Global Center for Evidence Synthesis, Chandigarh, India; ^13^University Center for Research and Development, Chandigarh University, Mohali, Punjab, India; ^14^Medical Laboratories Techniques Department, Al-Mustaqbal University, Hillah, Babil, Iraq; ^15^Department of Paediatrics, Dr. D. Y. Patil Medical College, Hospital and Research Centre, Dr. D. Y. Patil Vidyapeeth, Pune, Maharashtra, India; ^16^Department of Public Health Dentistry, Dr. D.Y. Patil Dental College and Hospital, Dr. D. Y. Patil Vidyapeeth, Pune, Maharashtra, India; ^17^IES Institute of Pharmacy, IES University, Bhopal, Madhya Pradesh, India; ^18^New Delhi Institute of Management, New Delhi, India; ^19^KIIT School of Biotechnology, KIIT University, Bhubaneswar, India; ^20^Unit of Immunology and Chronic Disease, Institute of Environmental Medicine, Karolinska Institute, Stockholm, Sweden

**Keywords:** neurological disorders, BRICS, DALYs, incidence, mortality, gender, sex disparities, metabolic risks

## Abstract

**Background:**

Neurological disorders are a major global health concern, especially in BRICS nations (Brazil, Russia, India, China, South Africa), where demographic and socio-economic changes have amplified their impact. This study evaluates trends in incidence, prevalence, mortality, and Disability-Adjusted Life Years (DALYs) associated with neurological diseases in these countries from 1990 to 2021, focusing on sex disparities and key risk factors.

**Methods:**

Data were obtained from the Global Burden of Disease (GBD) 2021 database. Join point regression and Estimated Annual Percentage Change (EAPC) analyses were used to assess trends in neurological disease burden. Age-standardized rates for incidence, prevalence, and mortality were calculated, along with DALYs, and key risk factors were analyzed.

**Results:**

China showed the largest increase in incidence (7541.89 to 8031.37 per 100,000) and prevalence (26494.85 to 28534.79 per 100,000). Mortality increased in India (21.01 to 24.27 per 100,000) and South Africa (27.66 to 30.65 per 100,000), while China showed a decline (39.59 to 37.30 per 100,000). Brazil experienced a substantial rise in DALYs (1610.65 to 42024.59). Sex disparities showed higher DALY rates for females across all nations.

**Conclusion:**

The research highlights the rising burden of neurological disorders in BRICS nations, especially in China and Brazil due to aging populations and metabolic risks. It emphasizes the need for targeted interventions in India and South Africa, where increasing mortality rates and DALYs are concerning. Effective health policies should focus on early detection, managing metabolic risks, and implementing sex-specific strategies to address these issues.

## Introduction

According to the Global Burden of Diseases, Injuries, and Risk Factors (GBD) study, neurologic disorders are a major cause of morbidity and mortality worldwide (1, 2). Neurological disorders represent a major cause of disability across the globe, with their impact on the overall health burden steadily rising over time (3). In 2021, more than 3.4 billion individuals worldwide were affected by nervous system-related conditions, emphasizing the critical need for greater awareness and intervention (4). The GBD study highlights the significant morbidity and mortality linked with neurological disorders, emphasizing the high disability-adjusted life years (DALYs) attributed to these conditions (5). Among the world’s emerging economies, the BRICS nations stand out due to their rapidly changing socio-economic, demographic, and epidemiological landscapes (6). These nations not only account for a substantial portion of the world’s population but also reflect diverse health outcomes shaped by varying healthcare systems, environmental exposures, and public health policies (7). The GBD study classifies neurological disorders to include communicable neurological diseases, stroke, headaches, neurodegenerative conditions, demyelinating diseases, cancers of the brain and central nervous system (CNS), along with a category for other less common neurological disorders (1, 2, 5). They pose a unique challenge within these countries, where the demographic shift toward aging populations is coupled with rising rates of non-communicable diseases (NCDs) (5, 8). These disorders are leading causes of disability and contribute heavily to the global burden of disease, particularly in aging populations (9). Tackling the burden of neurological NCDs is integral to achieving Sustainable Development Goal (SDG) 3, which aims to ensure healthy lives and promote well-being for all (10). Effective prevention, management, and equitable access to healthcare for these conditions are critical for reducing mortality and improving overall health outcomes.

However, there is a notable gap in comprehensive research that specifically addresses the neurological disease burden in BRICS nations (9). This study aims to provide a comprehensive examination of the epidemiological trends of neurological diseases in BRICS nations, focusing on incidence, prevalence, mortality, and DALYs. Additionally, it will analyze the influence of sex disparities and metabolic risk factors on these trends. By understanding these patterns, we can better inform public health strategies to reduce the burden of neurological diseases in these rapidly evolving regions. The findings will offer valuable insights for policymakers and healthcare professionals, supporting the development of more effective interventions and public health policies to manage and mitigate the impact of neurological diseases on global health.

## Methods

### Data sources

The data used in this study were obtained from the GBD 2021 database, an extensive and internationally recognized resource managed by the Institute for Health Metrics and Evaluation (IHME). GBD data is publicly accessible through the IHME’s online platform.^1^ Database provides estimates of incidence, prevalence, mortality, and DALYs for a wide range of diseases, including neurological disease. The data are standardized and adjusted for demographic factors, which makes them particularly suitable for cross-national comparisons like those in this study. The GBD study uses a variety of data sources, including national surveys, hospital records, and vital registration systems, to ensure comprehensive and accurate estimates. It also incorporates advanced statistical modeling techniques, such as Bayesian methods, to account for uncertainties and generate 95% Uncertainty Intervals (UI) for all estimates. The rigorous methodology and extensive coverage of the GBD database make it one of the most reliable sources for analyzing global health trends over time.

### Statistical analysis

#### Join point regression analysis

To evaluate trends over time, we employed join point regression analysis using Join point software version 5.0. This method identifies points where significant changes in trend occur and calculates the Annual Percentage Change (APC) for various time segments (11, 12). Join point regression was used to analyze trends and identify specific periods of significant change in each country’s neurological disease metrics. These metrics provided a detailed understanding of the shifting epidemiological burden across the BRICS nations. In addition to the APC, age-standardized rates for incidence were calculated for each country. We also compared the DALYs rates between males and females to assess sex disparities in neurological disease burden. Data were analyzed with 95% UI to account for variability and estimate the precision of the results.

### Estimated annual percentage change (EAPC) analysis

To quantify the long-term trends in incidence, prevalence, mortality, and Years DALYs of neurological diseases across BRICS nations from 1990 to 2021, we calculated the EAPC. The EAPC is a summary measure used to describe the average rate of change in age standardized rates (ASRs) over a specified time period. EAPC provides insight into whether the neurological disease burden has been increasing or decreasing over time, and how rapidly these changes are occurring. This metric is derived from the log linear regression model, where the natural logarithm of the ASR is regressed on calendar year. An EAPC greater than 0 indicates an increasing trend, while an EAPC less than 0 indicates a decreasing trend. EAPC estimates were calculated using Join point software version 5.0, which allowed for the identification of significant changes in trend segments. The EAPC was reported alongside 95% Confidence Intervals (CIs) to assess the precision of these estimates, and significance was determined if the CI did not include zero. This approach enabled a detailed examination of the temporal shifts in neurological disease burden across different countries and periods, providing a nuanced understanding of regional trends.

## Results

### Incidence of neurologic diseases

**Figure 1:**
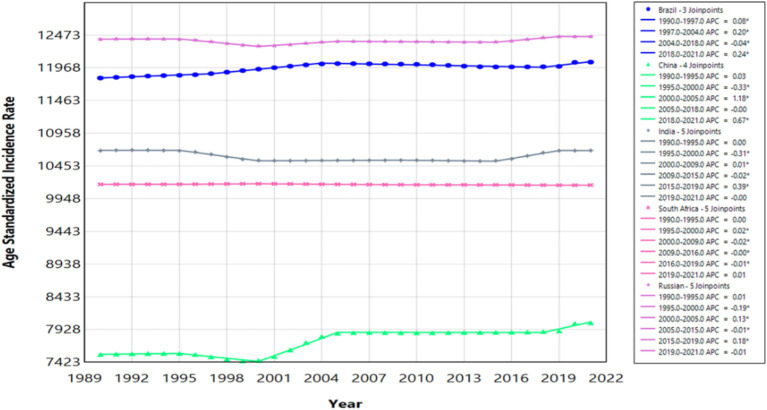
Join point regression analysis of neurologic disease trends in BRICS (Brazil, Russia, India, China, and South Africa) nations. * Indicates that the Annual Percent Change APC, AAPC is significantly different from zero at the alpha = 0.05 level. Age Standardized Incidence Rate (ASIR), Age Standardized Mortality Rate (ASPR).

**Figure 2:**
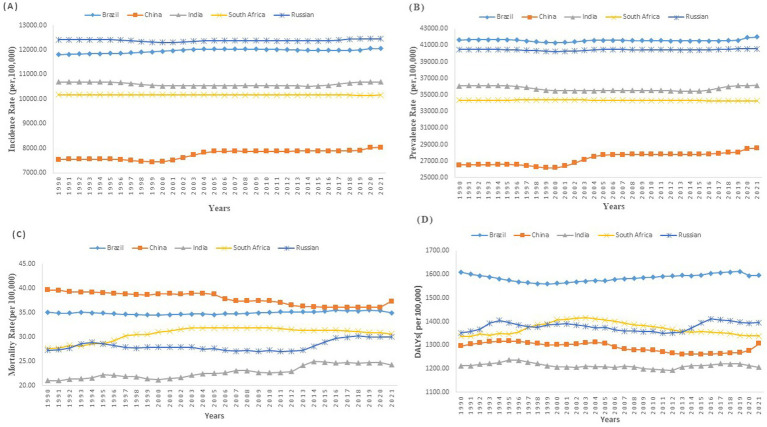
Temporal trends in age-standardized neurologic disease incidence, prevalence, mortality, and DALYs rates across BRICS (Brazil, Russia, India, China, and South Africa) nations (1990—2021) **(A)** Age-standardized Incidence Rate of Neurologic disease **(B)** Age-standardized Prevalence Rate of Neurologic disease **(C)** Age-standardized Mortality Rate of Neurologic disease **(D)** Age-standardized DALYs Rate of Neurologic disease.

A comprehensive analysis of age-standardized incidence rates (ASIR) for neurological diseases from 1989 to 2021 across BRICS nations reveals significant variations. In China, the ASIR increased notably from 7,541.89 per 100,000 in 1990 to 8,031.37 in 2021, with significant surges between 2000 and 2005 (APC of 1.18) and from 2018 to 2021 (APC of 0.67) (Table 1 and Figure 1). Brazil also demonstrated growth in ASIR, from 11,809.54 per 100,000 in 1990 to 12,055.55 in 2021, with increases from 1997 to 2004 (APC of 0.20) and from 2018 to 2021 (APC of 0.24). In contrast, India displayed a decrease in ASIR from 10,688.29 per 100,000 in 1990 to 10,691.07 in 2021, marked by a sharp decline from 1995 to 2000 (APC of −0.31), a subsequent rise from 2015 to 2019 (APC of 0.39), and stabilization from 2019 to 2021 (APC of −0.00). Russia’s ASIR changed minimally from 12,407.23 in 1990 to 12,449.37 in 2021 (Figure 2), characterized by declines (APC of −0.19 from 1995 to 2000) and increases (APC of 0.18 from 2015 to 2019). South Africa’s ASIR remained nearly constant, slightly decreasing from 10,169.27 in 1990 to 10,156.46 in 2021 (APC of −0.01).

**Table 1:** Age-Standardized incidence, prevalence, mortality, and DALYs rates of neurologic diseases (per 100,000) across BRICS Nations in 1990 and 2021 with estimated annual percentage change (EAPC) and 95% Uncertainty Intervals (UI).

	ASIR/100,000			ASPR/100,00			ASMR/100,00			DALYs/100,000		
Countries	1990 (95% UI)	2021 (95% UI)	EAPC (95% UI)	1990 (95% UI)	2021 (95% UI)	EAPC (95% UI)	1990 (95% UI)	2021 (95% UI)	EAPC (95% UI)	1990 (95% UI)	2021 (95% UI)	EAPC (95% UI)
Brazil	11809.54 (10645.73 to 12977.02)	12055.55 (10753.17 to 13237.49)	0.06 (0.04 to 0.07)	41639.15 (38611.19 to 44677.62)	42024.59 (38968.48 to 45409.76)	0.01 (−0.01 to 0.02)	35.04 (14.47 to 79.76)	34.9 (15.07 to 75.98)	0.05 (0.03 to 0.08)	1610.65 (861.74 to 2684.65)	42024.59 (38968.48 to 45409.76)	0.05 (0.02 to 0.08)
Russian	12407.23 (11013.19 to 13790.11)	12449.37 (11023.1 to 13814.88)	0.01 (0 to 0.02)	40480.45 (37546.21 to 43650.72)	40570.8 (37740.05 to 43744.65)	0.01 (0 to 0.02)	27.29 (11.04 to 65.08)	30.02 (14.35 to 66.46)	0.18 (0.06 to 0.3)	1351.33 (811.36 to 2121.1)	40570.8 (37740.05 to 43744.65)	0.03 (−0.02 to 0.08)
India	10688.29 (9501.78 to 11781.09)	10691.07 (9505.43 to 11786.84)	−0.02 (−0.04 to 0)	36083.11 (33371.65 to 38867.62)	36139.57 (33450.01 to 38900.79)	−0.02 (−0.05 to 0.01)	21.01 (10.54 to 46.26)	24.27 (11.6 to 52.94)	0.56 (0.48 to 0.65)	1212.61 (690.71 to 1973.01)	36139.57 (33450.01 to 38900.79)	−0.03 (−0.06 to 0)
China	7541.89 (6692.8 to 8385.96)	8031.37 (7121.85 to 8914.18)	0.23 (0.19 to 0.27)	26494.85 (24465.8 to 28641.06)	28534.79 (26445.69 to 30919.82)	0.26 (0.22 to 0.3)	39.59 (16.15 to 91.48)	37.3 (14.57 to 88.39)	−0.33 (−0.38 to −0.29)	1296.9 (769.11 to 2132.6)	28534.79 (26445.69 to 30919.82)	−0.13 (−0.17 to −0.09)
South Africa	10169.27 (9007.58 to 11303.21)	10156.46 (8996.44 to 11303.9)	−0.01 (−0.01 to 0)	34352.02 (31644.14 to 37062.67)	34273.79 (31588.57 to 36960.8)	−0.01 (−0.01 to −0.01)	27.66 (11.37 to 63.65)	30.65 (13.81 to 69.54)	0.34 (0.22 to 0.46)	1337.29 (805.77 to 2079.24)	34273.79 (31588.57 to 36960.8)	−0.02 (−0.09 to 0.05)

### Prevalence of neurologic diseases

**Figure 3:**
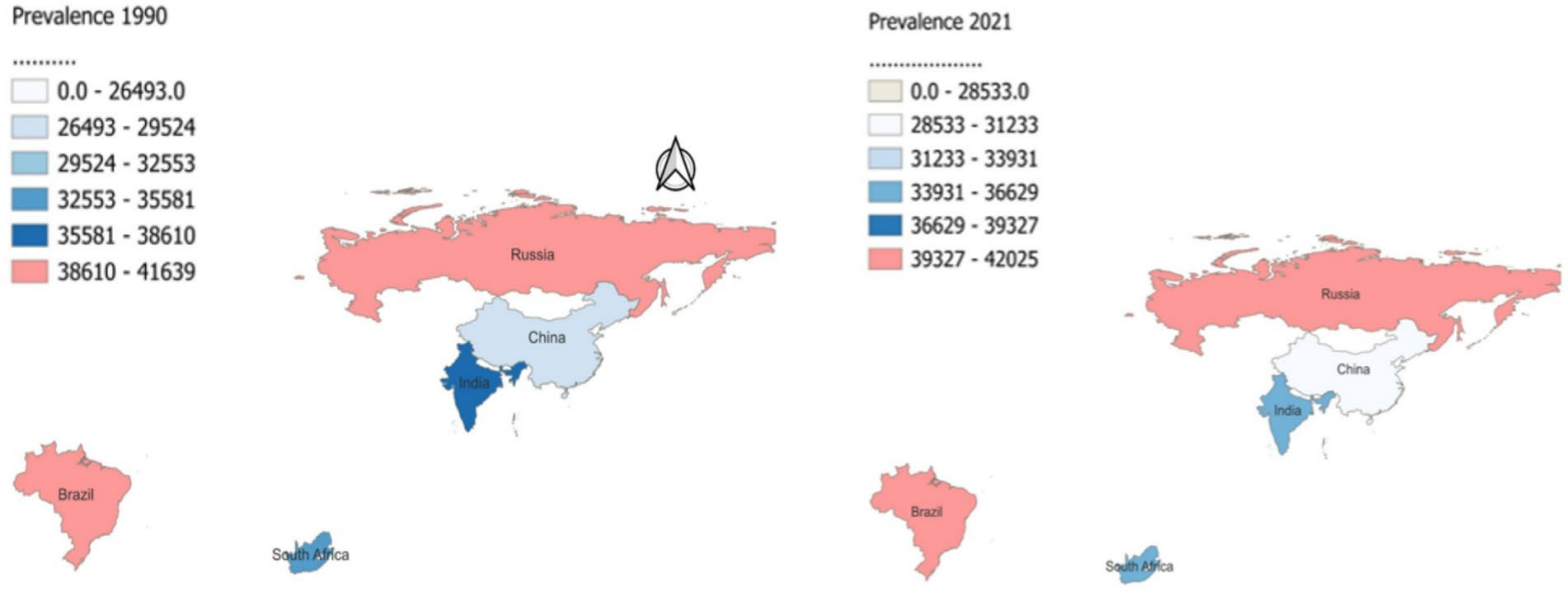
Age standardized prevalence rates of neurologic disease in Brazil, Russia, India, China, and South Africa in 1990 and 2021.

From 1990 to 2021, there were notable variations in the age-standardized prevalence rates (ASPR) of neurological diseases among the BRICS nations. Brazil observed a marginal increase in ASPR from 41,639.15 (95% UI: 38,611.19 to 44,677.62) to 42,024.59 (95% UI: 38,968.48 to 45,409.76), with an EAPC of 0.01 (95% UI: −0.01 to 0.02) (Table 1 and Figure 3). Similarly, Russia’s ASPR slightly rose from 40,480.45 (95% UI: 37,546.21 to 43,650.72) to 40,570.80 (95% UI: 37,740.05 to 43,744.65), with an EAPC of 0.01 (95% UI: 0 to 0.02). India’s ASPR remained nearly stable, with only a slight increase from 36,083.11 (95% UI: 33,371.65 to 38,867.62) to 36,139.57 (95% UI: 33,450.01 to 38,900.79), and an EAPC of −0.02 (95% UI: −0.05 to 0.01). China reported a more substantial increase, from 26,494.85 (95% UI: 24,465.80 to 28,641.06) to 28,534.79 (95% UI: 26,445.69 to 30,919.82), with an EAPC of 0.26 (95% UI: 0.22 to 0.3) (Table 1 and Figure 2). Conversely, South Africa experienced a minor decrease in ASPR from 34,352.02 (95% UI: 31,644.14 to 37,062.67) to 34,273.79 (95% UI: 31,588.57 to 36,960.80), with an EAPC of −0.01 (95% UI: −0.01 to −0.01).

### Mortality of neurologic diseases

Brazil’s age-standardized Mortality rates (ASMR) remained relatively stable, showing a minimal decrease from 35.04 per 100,000 (95% UI: 14.47 to 79.76) in 1990 to 34.9 per 100,000 (95% UI: 15.07 to 75.98) in 2021, with an EAPC of 0.05 (95% UI: 0.03 to 0.08) (Table 1 and Figure 2). Russia displayed a slight increase in ASMR from 27.29 per 100,000 (95% UI: 11.04 to 65.08) to 30.02 per 100,000 (95% UI: 14.35 to 66.46), with an EAPC of 0.18 (95% UI: 0.06 to 0.3).

India’s ASMR increased from 21.01 per 100,000 (95% UI: 10.54 to 46.26) to 24.27 per 100,000 (95% UI: 11.6 to 52.94), experiencing the highest EAPC among the BRICS at 0.56 (95% UI: 0.48 to 0.65). Conversely, China experienced a decline in ASMR from 39.59 per 100,000 (95% UI: 16.15 to 91.48) to 37.3 per 100,000 (95% UI: 14.57 to 88.39), with a negative EAPC of −0.33 (95% UI: −0.38 to −0.29). South Africa also saw an increase in ASMR from 27.66 per 100,000 (95% UI: 11.37 to 63.65) to 30.65 per 100,000 (95% UI: 13.81 to 69.54), with an EAPC of 0.34 (95% UI: 0.22 to 0.46).

### DALYs of neurologic diseases

**Figure 4:**
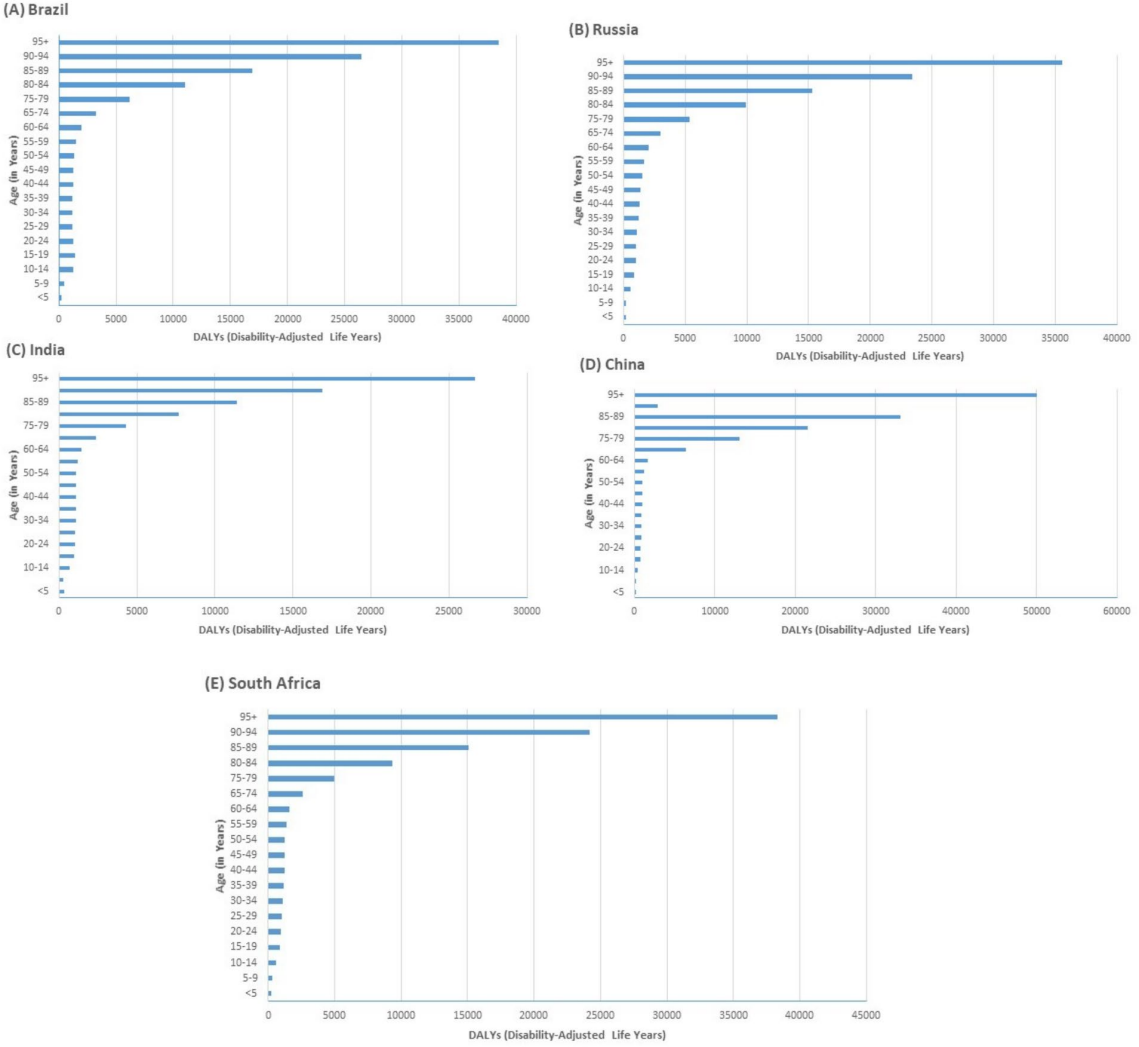
Disability-Adjusted Life Years (DALYs) distribution by age group in Brazil, Russia, India, China, and South Africa in 2021.

From 1990 to 2021, DALYs rates for neurological diseases across the BRICS nations exhibited modest changes. Brazil and Russia both saw slight increases in DALY rates. Brazil’s DALYs shifted from 1,610.65 per 100,000 (95% UI: 861.74 to 2,684.65) in 1990 to 1,596.31 in 2021, with an EAPC of 0.05 (95% UI: 0.02 to 0.08) (Table 1 and Figure 2). Russia experienced a rise from 1,351.33 per 100,000 (95% UI: 811.36 to 2,121.1) to 1,395.4, with an EAPC of 0.03 (95% UI: −0.02 to 0.08). Conversely, India, China, and South Africa observed slight declines. India’s DALYs marginally decreased from 1,212.61 per 100,000 (95% UI: 690.71 to 1,973.01) to 1,205.57, reflecting a negative EAPC of −0.03 (95% UI: −0.06 to 0). China reported a more notable decline in DALY rates from 1,296.9 per 100,000 (95% UI: 769.11 to 2,132.6) to 1,306.14, with a negative EAPC of −0.13 (95% UI: −0.17 to −0.09). South Africa saw a very minor reduction from 1,337.29 per 100,000 (95% UI: 805.77 to 2,079.24) to 1,338.49, with an EAPC of −0.02 (95% UI: −0.09 to 0.05). For the age-related DALYs, Brazil’s DALYs ranged from 226.13 for those under 5 to 38,489.06 for those aged 95 and above. Russia showed a similar progression from 231.74 to 35,542.74. In India, the range was from 323.87 to 26,671.56, while China’s figures spanned from 139.07 to 49,987.36, marking the highest increase (Figure 4). South Africa’s DALYs also increased significantly from 267.21 to 38,326.89.

### Sex disparities in neurologic diseases

The age-standardized DALY rates for neurological diseases in 2021 show notable sex disparities across BRICS nations. In Brazil, males had a DALY rate of 1,400.06 (95% UI: 816.37 to 2239.8) per 100,000, while females had a higher rate of 1,773.77 (95% UI: 3057.96 to 878.89), with a male-to-female ratio of 0.79 (Table 2). Similar patterns were observed in other BRICS countries, where the male-to-female ratio ranged from 0.75 in Russia to 0.85 in South Africa.

**Table 2:** BRICS countries age standardized DALY Rates (per 100,000) of male and female individuals and the male to female ratio in 2021 for neurologic diseases.

Country	Male (95% UI)	Female (95% UI)	Male to female ratio
Brazil	1400.06 (816.37 to 2239.8)	1773.77 (3057.96 to 878.89)	0.79
Russian	1177.4 (781.8 to 1775.42)	1579.01 (2519.92 to 924.53)	0.75
India	1084.93 (628.47 to 1766.34)	1323.3 (2274.62 to 683.51)	0.82
China	1161.99 (712.91 to 1866.65)	1434.96 (2374.93 to 753.88)	0.81
South Africa	1210.56 (787.36 to 1807.85)	1418.56 (2305.62 to 824.19)	0.85

DALY, disability adjusted life year; UI, Uncertainity Interval.

### Risk factors of neurologic diseases

**Figure 5:**
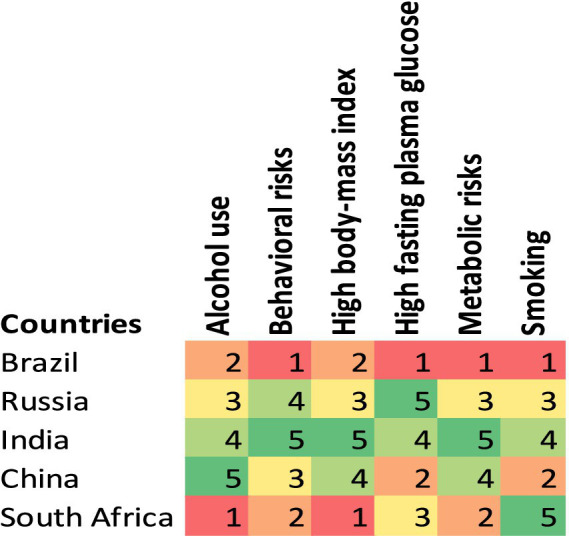
A heat map analysis: ranking of health risk factors in BRICS nations, 2021.

The impact of various risk factors on DALYs across BRICS nations demonstrated significant differences by sex and country. Alcohol use varied considerably, with Brazil showing a notable impact on males at 2.33% (95% UI: 1.2 to 4.04) compared to females at 0.51% (95% UI: 0.21 to 0.99), while South Africa reported the highest impact on males at 3.94% (95% UI: 2.32 to 6.32) (Table 3). Behavioral risks predominantly affected males more, as seen in Russia where the impact was 2.86% (95% UI: 1.51 to 4.99) for males versus 0.63% (95% UI: 0.37 to 1.03) for females. Metabolic risks also presented significant disparities, particularly in South Africa, where the impact on females reached up to 9.03% (95% UI: −1.15 to 24.18), compared to 5.68% for males. High fasting plasma glucose was another substantial risk, with China reporting 5.25% (95% UI: 0.38 to 12.29) for females and 4.68% for males. This heatmap analysis reveals distinct health risk profiles for BRICS nations in 2021 (Figure 5). In 2021, Brazil’s most critical health risk factors were identified as behavioral risks, high fasting plasma glucose, metabolic risks, and smoking. In contrast, South Africa’s primary health risk factors were alcohol use and high body mass index (BMI). Both countries reported a risk score of 1 for these factors.

**Table 3:** Percentage of DALYs attributable to risk factors of neurologic diseases in BRICS, 2021.

	Both sexes % (95% UI)	Males % (95% UI)	Females % (95% UI)
Brazil			
Alcohol use	1.29 (0.63 to 2.43)	2.33 (1.2 to 4.04)	0.51 (0.21 to 0.99)
Behavioral risks	2.32 (1.19 to 3.84)	3.65 (2.03 to 5.64)	1.37 (0.61 to 2.52)
High body-mass index	3.38 (−0.93 to 11.69)	3 (−0.74 to 10.03)	3.55 (−0.97 to 12.91)
High fasting plasma glucose	4.89 (0.33 to 12.31)	4.99 (0.32 to 12.06)	4.76 (0.33 to 12.53)
Metabolic risks	7.57 (−0.3 to 20.96)	7.35 (−0.17 to 19.4)	7.59 (−0.36 to 22.06)
Smoking	1.03 (0.32 to 2.33)	1.32 (0.34 to 3.12)	0.86 (0.3 to 1.79)
Russia			
Alcohol use	0.75 (0.39 to 1.23)	1.36 (0.73 to 2.19)	0.35 (0.16 to 0.64)
Behavioral risks	1.39 (0.81 to 2.28)	2.86 (1.51 to 4.99)	0.63 (0.37 to 1.03)
High body-mass index	3.8 (−1.22 to 12.36)	2.95 (−0.79 to 9.72)	3.92 (−1.29 to 13.14)
High fasting plasma glucose	3.27 (0.2 to 7.92)	3.26 (0.2 to 7.96)	3.11 (0.19 to 7.6)
Metabolic risks	6.47 (−0.92 to 18.1)	5.74 (−0.37 to 15.66)	6.42 (−1.06 to 18.25)
Smoking	0.64 (0.2 to 1.43)	1.49 (0.22 to 3.81)	0.28 (0.14 to 0.49)
India			
Alcohol use	0.76 (0.38 to 1.47)	1.58 (0.83 to 2.84)	0.06 (0.03 to 0.14)
Behavioral risks	1.21 (0.58 to 2.1)	2.35 (1.15 to 3.99)	0.32 (0.11 to 0.71)
High body-mass index	0.62 (−0.02 to 2.44)	0.41 (0 to 1.64)	0.77 (−0.04 to 2.92)
High fasting plasma glucose	4.29 (0.27 to 11.05)	4.11 (0.25 to 10.6)	4.35 (0.29 to 11.42)
Metabolic risks	4.77 (0.38 to 12.74)	4.42 (0.38 to 11.65)	4.95 (0.35 to 13.33)
Smoking	0.45 (−0.03 to 1.34)	0.77 (−0.17 to 2.45)	0.25 (0.06 to 0.62)
China			
Alcohol use	0.68 (0.35 to 1.19)	1.39 (0.74 to 2.29)	0.07 (0.03 to 0.13)
Behavioral risks	1.74 (0.63 to 3.45)	3.39 (1.02 to 6.94)	0.65 (0.27 to 1.23)
High body-mass index	1.85 (−0.16 to 6.44)	1.26 (−0.02 to 4.34)	2.21 (−0.28 to 7.61)
High fasting plasma glucose	5.08 (0.37 to 11.57)	4.68 (0.34 to 10.79)	5.25 (0.38 to 12.29)
Metabolic risks	6.62 (0.6 to 16.3)	5.72 (0.64 to 14.12)	7.09 (0.48 to 17.88)
Smoking	1.06 (−0.09 to 3)	2.01 (−0.5 to 6.01)	0.58 (0.2 to 1.18)
South Africa			
Alcohol use	2.11 (1.16 to 3.49)	3.94 (2.32 to 6.32)	0.68 (0.3 to 1.18)
Behavioral risks	2.49 (1.53 to 3.89)	4.43 (2.81 to 6.55)	1 (0.55 to 1.59)
High body-mass index	4.06 (−1.35 to 12.95)	2.55 (−0.65 to 8.46)	4.76 (−1.64 to 15.11)
High fasting plasma glucose	4.85 (0.28 to 12.04)	3.67 (0.2 to 9.6)	5.35 (0.32 to 13.04)
Metabolic risks	8 (−0.83 to 21.41)	5.68 (−0.18 to 15.75)	9.03 (−1.15 to 24.18)
Smoking	0.38 (0.1 to 0.89)	0.49 (0.04 to 1.41)	0.33 (0.11 to 0.68)

## Discussion

BRICS are emerging economies that collectively represent over 40% of the global population and account for a considerable portion of the global disease burden (13). Addressing the health challenges in these regions is crucial for global public health. While each country within the BRICS faces unique challenges, they also share common pressures, such as the dual burden of communicable and non-communicable diseases, healthcare disparities, and the need for more robust healthcare policies to address these issues. Neurological disorders are highly prevalent, with one in three individuals likely to experience such a condition during their lifetime. Between 1990 and 2016, these disorders were the leading cause of DALYs and the second largest cause of deaths globally, accounting for around nine million deaths each year (14). China stands out with a consistent increase in both incidence and prevalence of neurological diseases over the three-decade period. The rise in incidence rates, from 7541.89 per 100,000 in 1990 to 8031.37 in 2021. The escalation in incidence and prevalence indicates an enhanced capacity for recognition and diagnosis, possibly driven by demographic shifts such as an aging population and advancements in diagnostic technologies similar to results from other studies which shows that China’s rising prevalence of neurological diseases is closely tied to its aging population, while India’s increasing mortality rates align with studies highlighting the healthcare challenges faced in low and middle income countries (15). This trend is consistent with global data, which shows that neurological diseases tend to increase with age and is a major risk factor for numerous neurological diseases (16). As global populations age, the prevalence and incidence of age-related neurological conditions, such as Alzheimer’s and Parkinson’s, are expected to rise, especially in nations with large populations, such as those in the BRICS countries (17, 18). Neurological disorders like stroke, dementia, and Parkinson’s disease primarily impact older adults. The disease burden is particularly high among older adults, with mortality rates increasing with age, especially in those over 85 years (18, 19). The growing elderly population, along with the transition from communicable to non-communicable diseases, is driving the rise in the prevalence of these conditions (9, 16). In contrast, Brazil’s incidence rate only slightly increased from 11809.54 to 12055.55, which suggests relatively stable disease control but still high numbers, especially compared to other BRICS nations. The minimal rise could reflect a combination of better healthcare access and earlier detection of neurological conditions, yet it also indicates a persistently high baseline that may not have been significantly impacted by public health interventions. Similar trends are observed in Russia and South Africa, though with smaller increases, indicating that public health interventions in these countries might not have adequately addressed the root causes of neurological diseases.

Mortality trends show a mixed picture across the BRICS nations. China demonstrates a notable improvement in reducing mortality related to neurological diseases, with rates decreasing from 39.59 to 37.30 per 100,000. This decrease, coupled with a reduction in DALYs, suggests that China’s healthcare policies, particularly in reducing risk factors and improving access to early interventions, have been effective (20). These findings align with studies highlighting China’s progress in addressing non-communicable diseases through policy reforms, which have increased primary care utilization and improved health outcomes for individuals with NCDs (21). On the other hand, India and South Africa exhibited concerning trends. India’s mortality increased from 21.01 to 24.27 per 100,000, and DALYs surged significantly from 1084.93 to 1323.30, indicating a substantial rise in the burden of neurological diseases. This increase could be attributed to poor access to healthcare, late diagnoses, and the higher prevalence of risk factors such as metabolic diseases and behavioral risks. Similarly mortality increases were observed in South Africa, where DALYs grew markedly in the older population groups, highlighting significant healthcare gaps. The results also reveal a sex disparity in the burden of neurological diseases, with females consistently exhibiting higher DALYs compared to males across all BRICS nations. To address the higher DALY rates observed in women, BRICS nations should focus on developing sex-specific interventions that account for the increased burden of neurological diseases in females.

This sex disparity is likely driven by biological, social, and economic factors that differentially impact men and women, such as longer life expectancy for women and sex specific health risks. Similar patterns have been reported in global health studies, where women are often more affected by neurodegenerative diseases due to their longer lifespans. The role of steroid hormones is crucial, as they can influence the pathogenesis and progression of NDs. Understanding these mechanisms could lead to better prevention and treatment strategies (17, 22). In the case of neurodegenerative diseases, the increasing life expectancy and aging populations are leading to a growing percentage of individuals affected by these conditions. Epidemiological data show that Alzheimer’s disease is more common in women, while Parkinson’s disease and ALS (amyotrophic lateral sclerosis) are more common in men (22, 23). Neurological disorders affect global death rates and DALY, as well as quality-adjusted life years. Also, these disorders are the leading cause of disability and the second leading cause of death throughout the world (17, 24). Metabolic risks, including high fasting plasma glucose (HFPG) and body mass index (HBMI), were identified as the highest contributors to neurological disease DALYs, with 4.77% of DALYs attributable to metabolic risks. Our findings is consistent with the other global studies in which they documented HFPG as a crucial metabolic risk factor, with an increasing mortality rate associated with neurological diseases (25). This trend underscores the escalating impact of glucose dysregulation on neurological health outcomes. These findings highlight the critical role of metabolic health in neurological diseases. Similarly, our results align with observations that HBMI significantly elevates the risk of Neurological disease, emphasizing the urgent need for targeted weight management strategies in aging populations to mitigate this risk (25, 26). Behavioral risks, particularly alcohol use, were also significant, particularly in Brazil and South Africa, where the social and cultural acceptance of alcohol exacerbates public health challenges. The data suggest a need for comprehensive public health strategies targeting lifestyle modification, particularly in addressing metabolic and behavioral risk factors. The variability in DALY distribution across different countries further indicates that demographic factors play a crucial role, particularly in the highest-burdened age groups (90–94 and 95+). China consistently reports the highest DALYs across all older age groups, suggesting a need for greater healthcare resources and enhanced geriatric care in this country (18, 27). Brazil, South Africa, and Russia also exhibit significant DALYs in the 90–94 and 95+ age groups, highlighting the demand for preventive health measures to mitigate the growing burden in aging populations. India, while having lower DALYs than the other countries, still experiences a notable burden in the 95+ age group, pointing to the need for expanded healthcare services targeting this demographic. The sharp decline in DALYs among younger cohorts, such as the 80–84 and 60–64 age groups, particularly in Brazil and India, signals an opportunity to implement early intervention strategies to reduce the long-term health burdens. Between 1990 and 2021, changes in the age structure significantly contributed to the rise in DALYs for NCDs, with neurological disorders among the most affected (28). Public health strategies should focus on older populations in these countries, prioritizing resource allocation for geriatric care, preventive services, and infrastructure improvements to address the unique challenges posed by demographic shifts. As the elderly population grows, so will the demand for treatment, rehabilitation, and support services for neurological disorders, further intensifying financial pressures. Unlike other NCDs, such as heart disease, cancer, and diabetes, many neurological disorders have fewer known modifiable risk factors. This emphasizes the need for increased investment in translational research to uncover additional risk factors and develop targeted prevention and treatment strategies (29). Despite these findings, this study has several limitations that should be considered when interpreting the results. This study, reliant on secondary data analysis of the GBD data, acknowledges inherent limitations. While secondary data analysis facilitates extensive epidemiological assessments across different populations and periods, it depends on the original data’s quality and completeness. Specifically, variations in mortality reporting, notably in India, might introduce biases affecting disease burden assessments and policy formulation. The wide 95% UIs in many estimates also suggest substantial variability in measuring risk factors for neurological disorders, requiring careful interpretation of these trends. Nonetheless, secondary data analysis remains invaluable for understanding global health patterns and informing public health strategies. Future research should refine these methods and integrate genetic insights with economic analysis to develop targeted interventions for the unique challenges faced by these countries.

## Conclusion

The escalating burden of neurological diseases within BRICS nations, driven largely by demographic shifts and metabolic risk factors. The data reveals a marked increase in both incidence and prevalence, especially in China and Brazil, as aging populations grapple with metabolic health challenges. Alarming trends in mortality rates in India and South Africa further emphasize the pressing need for enhanced healthcare strategies. Additionally, the study highlights notable sex disparities, with females experiencing higher disability rates, pointing to a critical need for gender-specific health interventions. The significant role of high fasting plasma glucose as a metabolic risk factor across these populations underscores the importance of public health initiatives aimed at lifestyle adjustments and metabolic risk management. Addressing the growing neurological disease burden calls for the implementation of robust public health policies focused on early detection, effective risk management, and tailored interventions.

## Data Availability

Publicly available datasets were analyzed in this study. This data can be found at: http://ghdx.healthdata.org/gbd-results-tool.

## Footnotes

^1^ http://ghdx.healthdata.org/gbd-results-tool
